# Catechol-Based Antimicrobial Polymers

**DOI:** 10.3390/molecules26030559

**Published:** 2021-01-21

**Authors:** Seyedehfatemeh Razaviamri, Kan Wang, Bo Liu, Bruce P. Lee

**Affiliations:** Department of Biomedical Engineering, Michigan Technological University, Houghton, MI 49931, USA; srazavia@mtu.edu (S.R.); kanwang@mtu.edu (K.W.)

**Keywords:** catechol, polymer, antimicrobial, reactive oxygen species

## Abstract

Catechol is a key constituent in mussel adhesive proteins and is responsible for strong adhesive property and crosslinking formation. Plant-based polyphenols are also capable of chemical interactions similar to those of catechol and are inherently antimicrobial. This review reports a series of catechol-based antimicrobial polymers classified according to their antimicrobial mechanisms. Catechol is utilized as a surface anchoring group for adhering monomers and polymers of known antimicrobial properties onto various types of surfaces. Additionally, catechol’s ability to form strong complexes with metal ions and nanoparticles was utilized to sequester these antimicrobial agents into coatings and polymer matrices. During catechol oxidation, reactive oxygen species (ROS) is generated as a byproduct, and the use of the generated ROS for antimicrobial applications was also introduced. Finally, polymers that utilized the innate antimicrobial property of halogenated catechols and polyphenols were reviewed.

## 1. Introduction

Infection associated with microorganisms such as bacteria, viruses, fungi, or parasites results in more death worldwide when compared with other causes [1,2]. To date, there are many different strategies to prevent bacterial growth and infection. The most widely used antimicrobial strategy is the use of small antimicrobial molecules that are broadly applied such as antibiotics, antiseptics, disinfectants, and preservatives. However, overreliance on the use of these compounds has resulted in the formation of drug-resistant microorganisms due to their ability to rapidly mutate [3,4]. For instance, *Pseudomonas aeruginosa* and *Staphylococcus aureus* are resistant to many antibiotics [5].

Challenged by the ongoing threats from antibiotic-resistant microorganisms, polymers with intrinsic antimicrobial properties have received increased interest in both the academia and the industry [6,7]. Antimicrobial polymers are either functionalized with antimicrobial agents [8] or possess innate antimicrobial properties [9,10,11]. There are several categories of antimicrobial polymers, which include cationic polymers [12,13], polymers that mimic natural peptides [14,15,16,17], halogenated polymers [15,18], and polymers containing metal ions or nanoparticles (NPs) [19]. Antimicrobial polymers can slow or inhibit the growth of drug-resistant strains [9,10] and present high antimicrobial efficacy due to the various antimicrobial modes and polymeric structures [4]. Additionally, these antimicrobial polymers are promising materials with less toxicity to the human body, long-lasting activity, and higher environmental safety than the traditional disinfectants [20,21].

**Figure 1:**
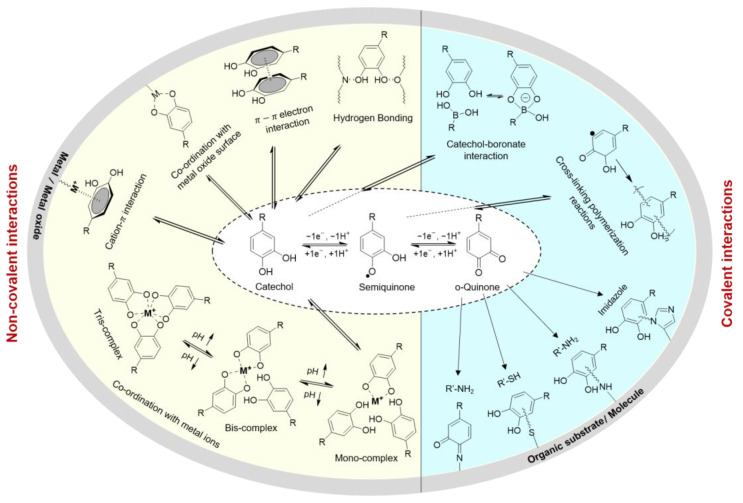
Possible interactions and reactions of catechol, semiquinone, and ortho-quinones. Noncovalent interactions include hydrogen bonding, π–π electron interaction, coordination with metal oxide surface, cation–π interaction, and coordination with metal ions. Covalent interactions include catechol–boronate complexation, polymerization, and irreversible bonding to organic substrates or molecules bearing, -thiol, -amine, and -imidazole functional groups.

Catechol and polyphenols are widely found in nature. Marine mussels secrete adhesive proteins that consist of a large abundance of 3,4-dihydroxyphenyl-l-alanine (DOPA), an amino acid with a catechol side chain [22,23,24]. The presence of catechol contributes to both the interfacial binding and curing of these adhesive proteins [25]. Catechol can participate in a wide range of reversible interactions (e.g., hydrogen bonding, π–π electron interaction, cation–π interaction, coordination with metal oxide surfaces and metal ions), and covalent bond formation (Figure 1) [23]. Incorporating catechol into the polymers imparts these materials with the chemical reactivity of catechol for designing adhesives, antifouling coatings, drug carriers, and antimicrobial polymers [26,27,28,29,30]. Similarly, plant-based polyphenols such as tannic acid (TA) and catechin exhibit intermolecular interactions and crosslinking capability resembling those of catechol [31,32,33]. While most scientists utilize these compounds predominantly as a surface anchoring group for promoting interfacial bonding, recent research indicated that catechol generates reactive oxygen species (ROS) as a byproduct during catechol oxidation [34]. ROS has been demonstrated to function as an effective, broad-spectrum biocide in many industrial and biomedical applications [35,36]. Additionally, catechol chemically modified with a halogen [37] and polyphenols such as TA, curcumin, catechin, and procyanidin [38,39,40] are innately antimicrobial.

This review focuses on catechol-based antimicrobial polymers. First, the use of catechol moieties as a surface anchoring group to immobilize antimicrobial polymers is reviewed. Then, the use of catechol-modified polymers to sequester metal ions or NPs is introduced. Next, the ability for catechol to generate antimicrobial levels of ROS is introduced. Finally, the antimicrobial activity of halogenated catechol and polyphenols is reviewed.

## 2. Catechol-Modified Polymers with Innate Antimicrobial Properties

Catechol can be incorporated into polymer chains by copolymerizing catechol-containing monomers with plant-based antimicrobial monomers or cationic monomers to prepare robust and biocompatible antimicrobial polymers and coatings [26,41]. Alternatively, catechol can be tethered to cationic polymers with known antimicrobial properties to synthesize antimicrobial polymers. In this strategy, catechol serves as the surface anchoring group to adhere these antimicrobial polymers onto various types of surfaces.

**Figure 2:**
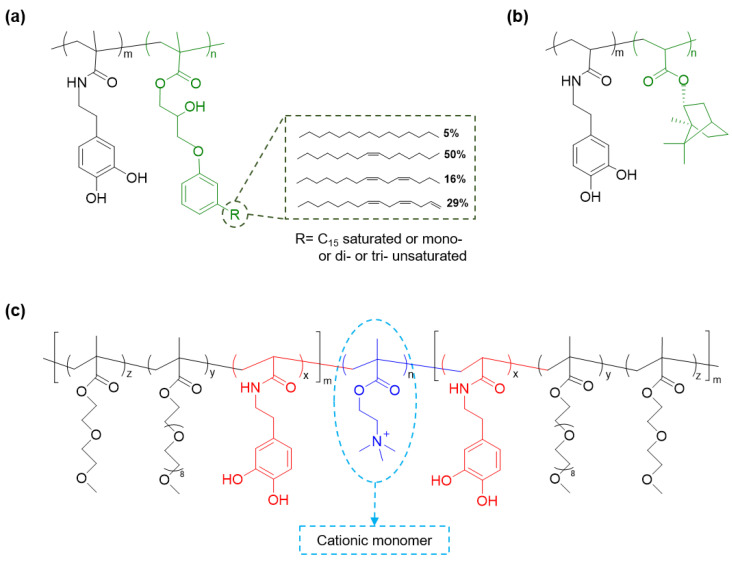
Chemical structures of polymers functionalized with catechol-based and antimicrobial monomers (**a**) P(DMA-*co*-HCPM), (**b**) P(DMA-*b*-BA), and (**c**) P(MEO_2_MA-*co*-OEGMA-*co*-DAA)-*b*-PMETA-*b*-P(MEO_2_MA-*co*-OEGMA-*co*-DAA).

Monomers with cardanol side chains, 2-hydroxy-3-cardanylpropyl methacrylate (HCPM), were copolymerized with dopamine methacrylamide (DMA) to prepare an antimicrobial polymer, P(DMA-*co*-HCPM) (Figure 2a) [40]. Cardanol can be obtained from cashew nut shell liquid and has previously demonstrated antimicrobial property [42,43]. The P(DMA-*co*-HCPM)-coated polysulfone membranes exhibited excellent antibacterial activities against *Escherichia coli* and *S. aureus*, demonstrating a higher than 90% killing efficiency. Similarly, borneol is a natural plant-based antibiotic [44], and borneol-containing polymers demonstrated excellent antibacterial activities [45]. However, these polymers do not have a surface anchoring moiety to form stable coatings [46]. Block copolymers of poly(DMA) and poly (borneolacrylate) (P(DMA-*b*-BA)) (Figure 2b) demonstrated remarkable and long-lasting antibacterial properties against *E. coli* and *S. aureus* [47]. P(DMA-*b*-BA) coatings showed robust adhesion and bactericidal properties on different surfaces such as silicon, silica, stainless steel (SS), cotton fabric, commercial gauze, and alumina.

Cationic polymers can kill pathogens by attacking their negatively charged cell walls and exhibit excellent antimicrobial properties [12]. The cationic monomer 2-(methacryloyloxy)-ethyl] trimethylammonium iodide (META) was copolymerized with polyethylene glycol (PEG) and catechol-based monomers to create a triblock copolymer poly{[2-(2-methoxyethoxy)ethyl methacrylate]-*co*-[oligo(ethylene glycol) methacrylate]-*co*-(*N*-3,4-dihydroxyphenethyl acrylamide)}-*b*-poly{[2-(methacryloyloxy)ethyl] trimethylammonium iodide}-*b*-poly{[2-(2-methoxyethoxy)ethyl methacrylate]-*co*-[oligo(ethylene glycol) methacrylate]-*co*-(*N*-3,4-dihydroxyphenethyl acrylamide)} (P(MEO_2_MA-*co*-OEGMA-*co*-DAA)-*b*-PMETA-*b*-P(MEO_2_MA-*co*-OEGMA-*co*-DAA)) (Figure 2c) [48]. This triblock copolymer can self-assemble to form a self-healing hydrogel, while effectively suppressing the growth of *E. coli* owing to the presence of cationic quaternary ammonium salt. Additionally, the incorporation of antifouling PEG prevented nonspecific cell attachment. Similarly, catechol was copolymerized with 2-(4-methylthiazol-5-yl) ethyl methacrylate (MTA) [49] and 2-(dimethylamino)ethyl methacrylate (DMAEMA) [50] to create cationic antimicrobial polymers.

Qiu et al. [51] showed that through the co-deposition of catechol and cationic polyethylenimine (PEI), *N*-alkylated PEI was grafted onto polypropylene microfiltration membranes (PPMs) at pH 8.5. Catechols are oxidized into quinone states in a weak alkaline condition, subsequently reacting with amino groups of PEI via Michael addition or Schiff base reaction. The modified membrane surface demonstrated 95% antibacterial efficiency against *S. aureus* and weak adherence of bacterial cells after 24 h of incubation.

## 3. Catechol-Based Polymers in Combination with Metal Ions and Nanoparticles

Metal ions such as silver, copper, zinc, gold, and titanium can kill bacteria by binding to cell membrane proteins, thus inhibiting vital enzymatic activities for cell growth and causing metabolic disruption that leads to cell death [52,53]. Catechol can form reversible complexes with these metal ions in a pH-dependent manner [54,55]. Additionally, catechol can reduce soluble metal ions to form NPs, thus functionalizing the NPs on the surface of the catechol-containing polymer [56,57,58]. This section reviews catechol-containing polymers that contained various metal ions and NPs. In these polymer systems, catechol functions as an adhesive moiety for surface bonding as well as sequestering the antimicrobial metal ions and NPs.

### 3.1. Silver Ions (Ag^+^) and Silver Nanoparticles (AgNPs)

**Figure 3:**
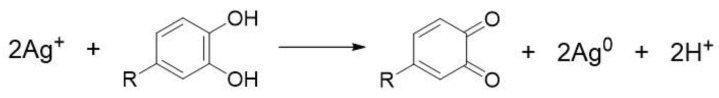
Silver nanoparticle (AgNP) preparation through the reduction of Ag^+^ by catechol as a reducing agent.

**Figure 4:**
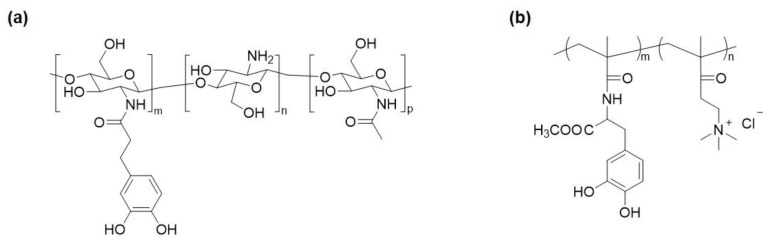
(**a**) Catechol-modified chitosan and (**b**) poly(mDOPA)-*co*-poly(DMAEMA^+^).

Silver and its compounds are the most used metal ions in creating antimicrobial polymers [5]. When silver is ionized in solution, the bactericidal active Ag^+^ binds to the proteins of cell walls and form complexes with the DNA and RNA of bacteria, leading to broad-spectrum antimicrobial activity [59,60]. Ag^+^ and AgNPs can be incorporated into copolymers, hydrogels, or coatings to create antimicrobial polymeric materials [30,61]. Catechol-containing polymers have been demonstrated to reduce water-soluble Ag^+^ to form AgNPs (Figure 3), effectively encapsulating the AgNPs into the polymer matrices. In this approach, catechol not only serves as the reducing agent but also stabilizes the in situ formed AgNPs. Huang et al. [62] prepared catechol-modified chitosan (Figure 4a), which reduced Ag^+^ in the form of silver nitrate (AgNO_3_) in solution to form an antimicrobial chitosan/AgNP composite. This composite exhibited remarkable antimicrobial performance at a very low dosage, with a minimum bactericidal concentration of 14 μg·mL^−1^ against *E. coli* and 25 μg·mL^−1^ against *S. aureus*. In another approach, O-carboxymethyl chitosan (CMC) was directly reacted with catechol and deposited onto polyethersulfone (PES) membranes to construct a coating loaded with AgNPs [63]. Carboxyl and amino groups in CMC captured Ag^+^, which was reduced to AgNPs by catechol moieties. Then PEG-based polyurethane (PU) was added to confer antifouling properties to the membrane. The chitosan/AgNP and CMC-Ag-PU composites can be deposited onto titanium and PES surfaces, respectively, by utilizing the strong adhesive and redox property of catechol [63,64]. Both surfaces exhibited strong antibacterial and antifouling properties against *E. coli* and *S. aureus*. Similarly, a copolymer with a cationic methacrylate bearing a quaternary ammonium group, 2-methacryloxyethyltrimethylammonium chloride (DMAEMA^+^), and a methacrylamide bearing DOPA group (poly(mDOPA)-*co*-poly(DMAEMA^+^)) (Figure 4b) was applied to fabricate an antimicrobial coating for SS [65]. This cationic polymer in combination with a polyanion, poly(styrene sulfonate), was deposited on the SS surface by electrostatic interaction. Both DOPA and poly(DMAEMA^+^) formed and stabilized bactericidal AgNPs. This coating showed excellent killing capability against *E. coli*. The antimicrobial Ag^+^ can be reloaded to replenish the antimicrobial coating. This approach utilized a one-pot preparation, which is more convenient than the layer by layer (LbL) deposition in which 45–60 bilayers are needed to have a comparable antimicrobial activity [66].

In addition to the catechol-based copolymers, diverse antimicrobial catechol-based hydrogels containing AgNPs were also reported. Le Thi et al. [67] described catechol-functionalized gelatin hydrogels loaded with AgNPs for enhanced antimicrobial activities. This composite hydrogel sustainably released Ag^+^ over a period of 14 days, which demonstrated the ability to inhibit the growth of both *E. coli* and *S. aureus* bacteria. Similarly, GhavamiNejad et al. [68] embedded AgNPs into a zwitterionic hydrogel copolymerized with DMA. This composite hydrogel exhibited strong antibacterial properties against Gram-negative (*E. coli*) and Gram-positive (*S. aureus* and *P. aeruginosa*) bacteria. Other monomers such as non-ionic, cationic, and anionic monomers can be used instead of zwitterionic monomers to fabricate AgNP-containing nanocomposite hydrogels.

There are limitations for using AgNPs in biomedical applications due to its potential for causing mammalian cell apoptosis and death [69,70]. Dopamine-conjugated polymers can be used to reduce the toxicity of AgNPs toward mammalian cells [71]. In this one-step approach, antimicrobial and biocompatible catechol-containing silver-carbon nanotube composites (AgNP-CNT) were produced. A catechol-containing heparin-mimetic polymer was used to convert Ag^+^ to AgNPs and anchor them onto the surface of the CNT composites. The composite coatings demonstrated a great antibacterial activity against *E. coli* and *S. aureus* with the killing efficiency of 77.3% and 81.2%, respectively. Interestingly, the shielding effects of the catecholic polymer coating and the bioactivity of the heparin-like polymer resulted in the improvement of the cytocompatibility of the antimicrobial nanocomposites and inhibited the direct cellular exposure to AgNPs.

Gan et al. [72] developed a plant-based hydrogel containing Ag-lignin NPs, pectin (P), and poly acrylic acid (PAA). Lignin possesses the reductive phenolic hydroxyl and methoxy groups, which can reduce Ag^+^ to AgNPs. The Ag-lignin NPs-P-PAA hydrogel displayed long-term adhesion, high toughness, and strong antimicrobial properties. The increased adhesive property was due to the continuous generation of the catechol from of lignin through a balanced redox reaction inside the hydrogel network. This hydrogel effectively inhibited *E. coli* (97%) and *Staphylococcus epidermidis* (98%). The antibacterial activities of NPs-P-PPA in vivo were confirmed in a rabbit model following the injection of *E. coli* suspension (1 mL, 10^5^ cells mL^−1^).

### 3.2. Other Metal Ions and Nanoparticles

**Figure 5:**
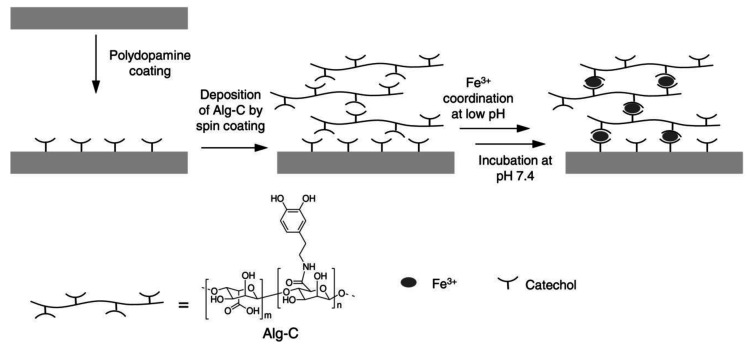
Alg-C/Fe^3+^ coating. Catechol-Fe^3+^-catechol interactions are the bridge to construct the antimicrobial multilayer film. Reprinted with permission from reference [74], copyright 2016 Wiley.

Iron ion (Fe^3+^) is widely found in mussel byssus along with catechol-containing proteins [73]. The catechol–Fe^3+^ interaction has been reported as a tool for developing an antimicrobial polymer film on a solid surface [74]. Alginate-functionalized with catechol (Alg-C) was deposited onto polydopamine (PDA)-coated substrate and Fe^3+^ was introduced as the crosslinker to construct a multilayered film (Figure 5). PDA was first described by the Messersmith lab [75] and is a facile method to form multifunctional coatings consisting of polymerized form of dopamine. The Alg-C/Fe^3+^ multilayered films prevented bacterial adhesion and films with a thickness greater than 10 nm demonstrated the ability to inhibit bacterial growth for over 24 h.

Siderophores are iron-chelating compounds, secreted by cells to gather iron from external sources [76]. Artificial catechol-containing siderophores conjugated with antimicrobial drugs displayed potent antimicrobial activity against multidrug-resistant bacteria. These catecholate siderophores form complex with Fe^3+^ and enter the microorganism via the corresponding siderophore-uptake pathway to deliver the antimicrobial drug to the bacterial cell. These drug conjugates exhibited strong antibacterial activities against Gram-negative bacteria such as *P. aeruginosa*, which is highly resistant to most of the existing antibiotics [77]. Some conjugates exhibited a minimum inhibitory concentration lower than 0.25 μg·mL^−1^ when treated against aminopenicillin-resistant strains [78].

Molybdenum trioxide (MoO_3_) NPs also demonstrated strong antimicrobial activity [56,57]. However, the application of MoO_3_ NPs is limited by their poor solubility in water. Catechol-containing polymers such as poly(dopamine methacrylamide-*co*-methoxyethyl acrylate), poly(dopamine methacrylamide), poly(ethyl methacrylate-*co*-dopamine methacrylamide), and poly (hydroxyethyl methacrylate-*co*-dopamine methacrylamide) were used to secure MoO_3_ NPs on surfaces [58]. These nanocomposite coatings not only killed *E. coli* and *Bacillus subtilis* after only 1 h of incubation, but they were also antimicrobial against the more antibiotic-resistant Gram-negative (*P. aeruginosa*) and Gram-positive (*Streptococcus pyogenes* and *S. epidermidis*) bacteria strains after 2 h of incubation. These coatings also demonstrated the ability to inhibit the growth of biofilms.

## 4. ROS-Releasing Catechol-Based Polymers

ROS are highly reactive molecules and free radicals derived from molecular oxygen [79]. ROS can degrade organic compounds [80,81,82], initiate free radical polymerization [83], and kill cells [84,85]. ROS kills cells by attacking and destroying proteins, lipids, and DNA, which makes ROS a potential solution for antimicrobial applications [86]. Catechol generates various types of ROS such as hydrogen peroxide (H_2_O_2_), superoxide (O_2_^−^), singlet oxygen (^1^O_2_), and hydroxyl radical (•OH) during oxidizing conditions such as autoxidation [34], chemical-induced oxidation [87], and metal ion-mediated oxidation [87,88].

**Figure 6:**
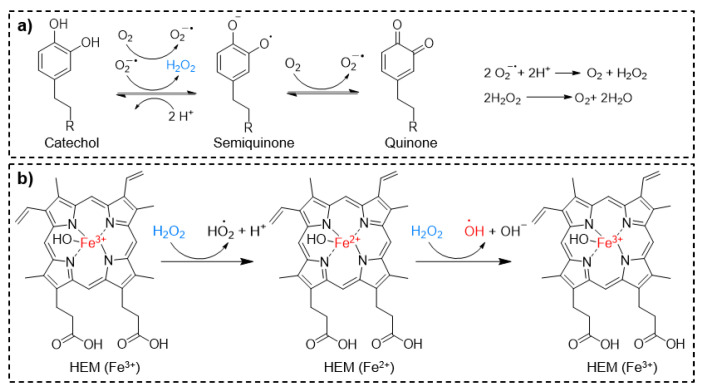
Schematics showing the mechanism of catechol oxidation and H_2_O_2_ generation (**a**) and H_2_O_2_ decomposition to generate •OH in the presence hematin (HEM) (**b**). Reproduced with permission from reference [90] copyright 2020 American Chemical Society.

H_2_O_2_ is generated as a byproduct during the autoxidation of catechol at a neutral to basic pH (Figure 6a) [34]. Catechol-modified microgels generated 1–5 mM of H_2_O_2_ over a period of 4 days as catechol autoxidized through simple hydration [28]. The H_2_O_2_ generated from these microgels completely prevented colony formation of both Gram-negative (*E. coli*) and Gram-positive (*S. epidermidis*) bacteria within 24 h and inactivated the infectivity of both enveloped bovine viral diarrhea virus (BVDV) and non-enveloped porcine parvovirus (PPV). By controlling the oxidation state of catechol, these microgels can be repeatedly activated (pH 7.4) and deactivated (pH 3.5) to generate antipathogenic levels of H_2_O_2_. These microgels do not contain the reactive ROS, and H_2_O_2_ is generated by converting molecular oxygen in the aqueous solution through catechol oxidation. This simple activation process enables the catechol-modified microgel to function as a lightweight and portable source of disinfectant.

H_2_O_2_ is not a very potent disinfectant and bacteria such as *Staphylococcus* secrete antioxidant enzymes such as catalase that decomposes H_2_O_2_ [89]. To further enhance the antimicrobial property of catechol-modified microgels, these microgels were further chemically modified with hematin (HEM), a porphyrin derivative that contains an Fe^3+^ ion (Figure 6b) [90]. Fe^3+^ can convert the generated H_2_O_2_ to •OH via a Fenton-like reaction process. •OH is also a highly reactive and strong oxidant with remarkable antimicrobial properties [91]. These microgels demonstrated faster and more effective antibacterial activities against both Gram-negative (*E. coli*) and Gram-positive (*S. epiermidis*) bacteria at concentrations of 10^6^ and 10^7^ CFU·mL^−1^, when compared to microgels that generated only H_2_O_2_ [90]. These microgels also reduced 99.997% and 99.97% infectivity of BVDV and PPV, respectively. However, •OH alone did not provide sufficient antimicrobial property due to its short half-life (10^−9^ s) [92]. To overcome this issue, the microgels were further modified with positively charged [2-(methacryloyloxy)ethyl] trimethylammonium chloride (METAC), which enhances the antibacterial performance of the microgel through electrostatic interactions between the positively charged microgels and the negatively charged pathogens [90].

**Figure 7:**
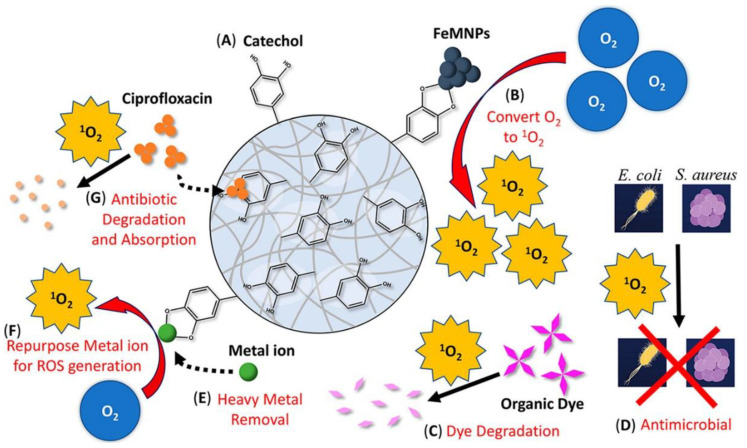
Multifunctional catechol-based microgel. Reprinted with permission from reference [88], copyright 2020 American Chemical Society.

Catechol generates O_2_^−^ in metal ion-mediated oxidation, which can be further converted into ^1^O_2_ by the metal ion [88,93]. Both O_2_^−^ and ^1^O_2_ are more reactive when compared to H_2_O_2_. When catechol-modified microgels were incubated in solutions containing up to 40 mM of various metal ions (e.g., Fe^2+^, Ni^2+^, Cu^2+^, Co^2+^) more than 85% of these metal ions were removed from the solution [88]. Most interestingly, these metal ions were repurposed to generate ROS for dye degradation. Similarly, ^1^O_2_ was produced by oxidizing catechol-modified microgel with iron magnetic nanoparticles (FeMNPs) instead of metal ions (Figure 7). Unlike autoxidation of catechol that occurs only at a basic pH, the ROS generation occurred over a wide range of pH (pH 3 to 9). The generated ^1^O_2_ killed 99% of *E. coli* after 24 h of incubation, degraded organic dyes, and removed the antibiotic ciprofloxacin from the solution. This simple mixture of catechol-modified microgel and FeMNPs can potentially be utilized as a portable source for on-demand generation of ROS for bioremediation and water purification.

**Figure 8:**
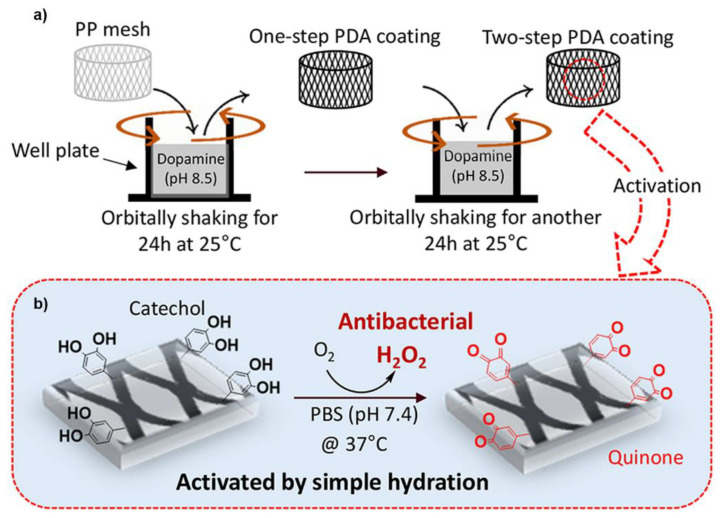
Schematic illustration of (**a**) catechol-based polydopamine (PDA) coating prepared by two-steps with gentle shaking and (**b**) the activation of the PDA-coated mesh to generate H_2_O_2_ by simply hydrating the mesh in the PBS with at pH 7.4. Reprinted with the permission of reference [29], copyright 2019 Kord Forooshani et al.

Catechols in PDA coating also demonstrated the ability to generate H_2_O_2_ [29]. However, to generate antimicrobial levels of H_2_O_2_, a two-step coating approach combined with gentle shaking was necessary (Figure 8). In the first coating step, a thick primer layer of PDA was coated onto the surface of polypropylene (PP) mesh utilizing an elevated level of dopamine (20 mg·mL^−1^). In the second step, a significantly lower concentration of dopamine (2 mg·mL^−1^) was applied for the formation and deposition of macroaggregates of PDA NPs formed in the solution. Shaking the solution during coating promoted gas exchange to increase molecular oxygen content in the reaction solution, which promoted catechol oxidation in creating a thicker PDA film. When the PDA-coated PP mesh was hydrated in a solution at pH 7.4, 200 μM of H_2_O_2_ was generated for over 48 h. The released H_2_O_2_ completely killed *E. coli* and reduced the log reduction value of *S. epidermidis* by 98.9% within 24 h. Furthermore, PDA was coated on to SS to reduce adhesion of *Psychrobacter cryohalolentis* [94].

## 5. Innate Antimicrobial Property of Halogenated Catechol and Polyphenols

### 5.1. Antimicrobial Halogenated Catechol

Halogenated phenols, such as triclosan and hexachlorophenol, can rupture and kill bacteria by deforming their cell walls, inhibiting their growth, and causing cytological damage [95]. Triclosan binds tightly to enoyl-acyl carrier protein reductase in complex with oxidized nicotinamide adenine dinucleotide (FabI/NAD^+^) to inhibit the synthesis of bacterial fatty acids and achieve a broad-spectrum antimicrobial effect [96,97]. The antimicrobial halogenated catechol also exists in nature. DOPA with a chloride-functionalized catechol side chain (Cl-DOPA) was extracted from a marine polychaete, *Phragmatopoma californica* [98]. PEG hydrogel chemically crosslinked using Cl-functionalized dopamine prevented *E. coli* adhesion rate by 20% [99].

**Figure 9:**
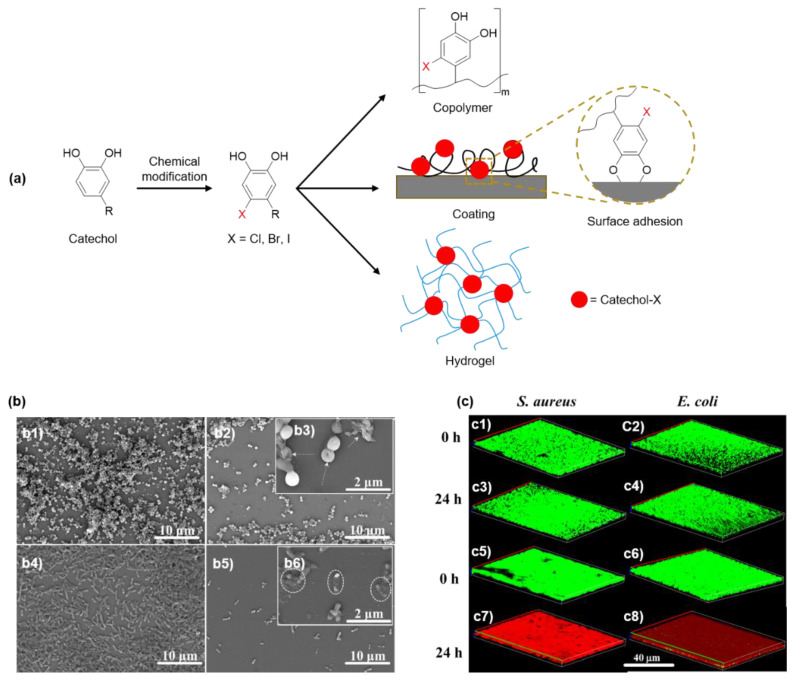
(**a**) Chemical structures of halogenated catechol, which was incorporated into copolymers, coatings, and hydrogels with antimicrobial property. (**b**) FE-SEM images of (b1) *S. aureus* grown after 24 h. (b2 and b3) *S. aureus* treated with DMA-Cl containing hydrogel for 24 h with the inset image showing magnified image of ruptured *S. aureus* and bacterial debris (white arrows). (b4) *E. coli* culture for 24 h. (b5 and b6) *E. coli* treated with DMA-Cl containing hydrogel for 24 h with the inset image showing magnified image of ruptured *E. coli* and bacterial debris (white circles). (**c**) Fluorescence images of LIVE/DEAD bacterial staining assay of *S. aureus* (c1 and c3) treated with catechol-free hydrogel and (c5 and c7) DMA-Cl-containing hydrogel after 0 and 24 h. Fluorescence images of LIVE/DEAD bacterial staining assay of *E. coli* (c2 and c4) treated with catechol-free hydrogel and (c6 and c8) DMA-Cl-containing hydrogel after 0 and 24 h. Live and dead cells are stained green and red, respectively. Reprinted from [37], copyright 2021, with permission from Elsevier.

Recently, our group prepared a series of DMA derivatives (chlorodopamine methacrylamide (DMA-Cl), bromodopamine methacrylamide (DMA-Br), and iododopamine methacrylamide (DMA-I)) modified with electron-withdrawing halogen substituents at the 6-position (Figure 9) [37]. These halogenated DMAs were incorporated into hydrogels, copolymers, and coatings through free-radical polymerization. The killing efficiency of halogenated DMA-containing polymers exhibited a 7 log reduction against *E. coli* and *S. aureus*. Most notably, DMA-Cl containing hydrogels effectively killed five multidrug-resistant (MDR) bacteria (methicillin-resistant *S. aureus*, vancomycin-resistant *enterococci*, multi-antibiotics-resistant *P. aeruginosa*, multi-antibiotics-resistant *Acinetobacter baumannii*, and carbapenem-resistant *Klebsiella pneumoniae*). All MDR bacteria were completely eradicated after 24 h of incubation. Additionally, these hydrogels also demonstrated the ability to kill bacteria in a biofilm while exhibiting low cytotoxicity. Interestingly, when the catechol side chain was protected with methoxy groups and rendered non-adhesive, the methoxy-protected catechol lost its antimicrobial activity. This indicated that the ability for catechol to adhere to the bacteria is critical for contact killing, which resulted in membrane disruption. Other halogenated catechol-based polymers such as chlorinated PDA (Cl-PDA) demonstrated a 5 log reduction in bacterial population against both *E. coli* and *S. aureus* [100].

### 5.2. Antimicrobial Polyphenols

**Figure 10:**
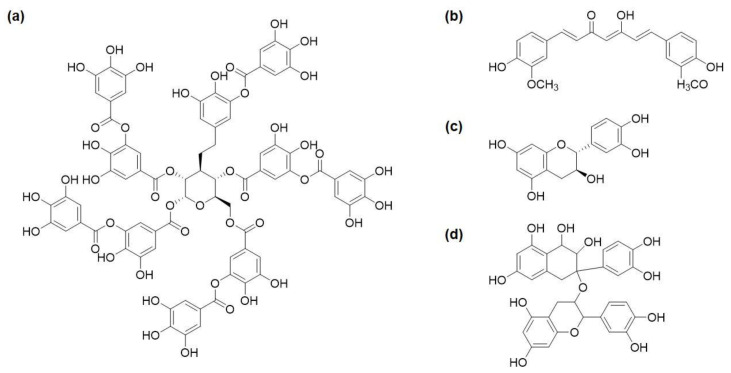
Chemical structures of antimicrobial polyphenols such as (**a**) TA, (**b**) curcumin, (**c**) catechin, and (**d**) procyanidin.

Polyphenols such as TA, curcumin, catechin, and procyanidin (Figure 10) exhibit innate antimicrobial properties due to the abundant phenolic hydroxyl groups, which can denature bacterial proteins and damage bacterial cell membranes [38,39,40]. TA has demonstrated antimicrobial effect on *S. aureus* [101]. The antibacterial activity of TA largely relies on the content of phenolic hydroxyl groups [102]. Sahiner et al. [103] prepared a crosslinked poly(TA) hydrogel. Under acidic conditions (pH 5.4), p(TA) hydrolyzed into gallic acid, the minimum inhibition concentration (MIC) value of p(TA) against *S. aureus* was 40 μL·mL^−1^. Under alkaline conditions (pH 9.0), p(TA) hydrolyzed and released TA, the MIC value of p(TA) against *S. aureus* was 10 μL·mL^−1^. Li and coworkers [104] prepared hemostatic microparticles by crosslinking TA, carboxymethyl chitosan, hyaluronic acid, and starch. When this composite material was added to the wound site, it promoted rapid hemostasis, and the released TA exhibited antimicrobial effects against both *E. coli* and *S. aureus*. A series of UV-curable antibacterial resins were synthesized by modifying TA with different amounts of glycidyl methacrylate (GMA) [105]. This resin achieved diameters of zone of inhibition as high as 19 mm against *E. coli* and *S. aureus*. However, with an elevated amount of GMA used to crosslink the resin, the resin lost antimicrobial property, indicating that the phenolic hydroxyl groups in TA played an important role in antibacterial activity [106].

Other natural polyphenols such as tea catechins, curcumin from *Curcuma longa*, and procyanidins from grape seeds exhibit anti-tumor, anti-inflammatory, antioxidant, anti-obesity, and antimicrobial properties [107,108,109,110,111,112]. For example, theaflavin digallate (TFDG), a poly-catechin, can directly inhibit cytoplasmic membrane proteins to achieve an antimicrobial effect [113]. The membrane glucose transporters’ activity decreased 40% after treatment with 62.5 mg·L^−1^ TFDG. Similarly, nanofibrous membranes constructed from curcumin-containing polymer demonstrated to be effective antimicrobial barriers with antimicrobial activity that lasted over 7 days [111]. Procyanidins can serve as an antimicrobial drug [114]. Procyanidins were loaded into sugarcane bagasse hydrogel and exhibited antibacterial effect against *S. aureus*. Finally, procyanidin-treated crepe de Chine silk showed excellent flame-retardant and antimicrobial properties [115]. The treated silk maintains more than 80% antimicrobial activity after repeated washing for more than 20 times.

## 6. Summary and Future Outlooks

The use of antimicrobial polymers has been extended to many different fields due to their improved quality and safety in comparison to traditionally used biocides. This article reviewed different strategies to create antimicrobial polymers utilizing catechol chemistry. The adhesive property of catechol was utilized to anchor antimicrobial polymers to impart surfaces with antimicrobial property. Additionally, the ability for catechol to bind to metal ions and reduce metal nanoparticles was utilized to sequester these antimicrobial ions and particles into coatings and polymer matrices. ROS is a broad-spectrum disinfectant and is generated as a byproduct during catechol oxidation. The process of inducing in situ catechol oxidation is a recent strategy utilized to create portable biomaterials with the ability for on-demand generation of ROS for antimicrobial application. Finally, halogenated catechols and natural polyphenols exhibit innate antimicrobial property.

While catechol and catechol-containing biomaterials have proven to be biocompatible in culture and in preclinical studies [116,117,118,119], cytotoxic compounds are incorporated in designing antimicrobial polymers. Ag^+^ can interfere with mammalian cell function through a competitive protein complexation and silver-containing polymers can damage mammalian cells [69,70]. Antimicrobial metal oxides such as zinc and titanium with improved biocompatibility can potentially be used instead of the cytotoxic Ag^+^ [120,121,122]. Similarly, halogenated catechol such as chlorocatechols had been demonstrated to be toxic to zebra fish, a model organism [123]. Additionally, iodine-modified catechol was also demonstrated to be cytotoxic when directly contacting fibroblasts [37]. To improve the biocompatibility of halogenated catechol, a temporary and pH-responsive protecting group such as boronic acid could potentially be incorporated [124,125]. The utilization of ROS is an attractive antimicrobial strategy due to its short half-life and biocompatible degradation products (i.e., water and oxygen) [35]. ROS is also a natural disinfectant generated as part of normal wound healing response [79]. However, elevated levels of ROS can destroy healthy tissues, retard wound healing, and induce tumor formation [126,127]. Silica nanoparticles that catalyze the degradation of ROS could potentially be incorporated to modulate the concentration of the released ROS [128].

One of the often-overlooked issues in designing catechol-based coating is the long-term stability of the surface-bound catechol. There have only been limited studies that characterized the performance of these coatings in the presence of biomolecules or cells, or in vivo for 7 days or longer [129,130]. Catechol forms both reversible and irreversible interfacial bonds depending on the surface type [22,23,24], and it is potentially feasible for catechol to detach from inorganic surfaces over time. Recently, in situ electrochemical oxidation was found to deactivate and detach catechol-containing adhesive that was adhered to a titanium surface [131]. As such, externally applied force and oxidative stress can potentially lead to catechol delamination. While synthetic mussel adhesive mimics predominantly utilize catechol for adhesion, mussel adhesive proteins utilize a combination of different amino acid residues (i.e., charged, hydrophobic, etc.) and intermolecular chemical interactions between multiple proteins to create adhesive plaques that bind tightly to the substrate surface [23]. Incorporation of diverse interfacial chemistries may be necessary to strengthen coating stability. Additionally, there is a potentially need for strategies that preserve the reduced and adhesive form of catechol so that the delaminated catechol may reattach. The incorporation of an antioxidant thiol functional group [132], acidic side chain for buffering local solution pH [133], and temporary protecting groups such as boronic acid [134] can be used to prevent catechol oxidation.
